# Supplementary material for: The role of neonatal kisspeptin in long-term social behavior in mammals
Source: Commun Biol. 2025 Jul 22;8:1085. doi: 10.1038/s42003-025-08478-x (PMC12284216; doi:10.1038/s42003-025-08478-x)
Supplement: Supplementary file 2 — Supporting Information [file 42003_2025_8478_MOESM2_ESM.pdf]

Supporting information

Graphical abstract

This graphical abstract was created using Biorender.com

Supplemental information

File name: Supporting information

Description: Supplementary figures 1-3 and captions.

File name: Supplementary Data

Description: The source data behind the graphs in the paper

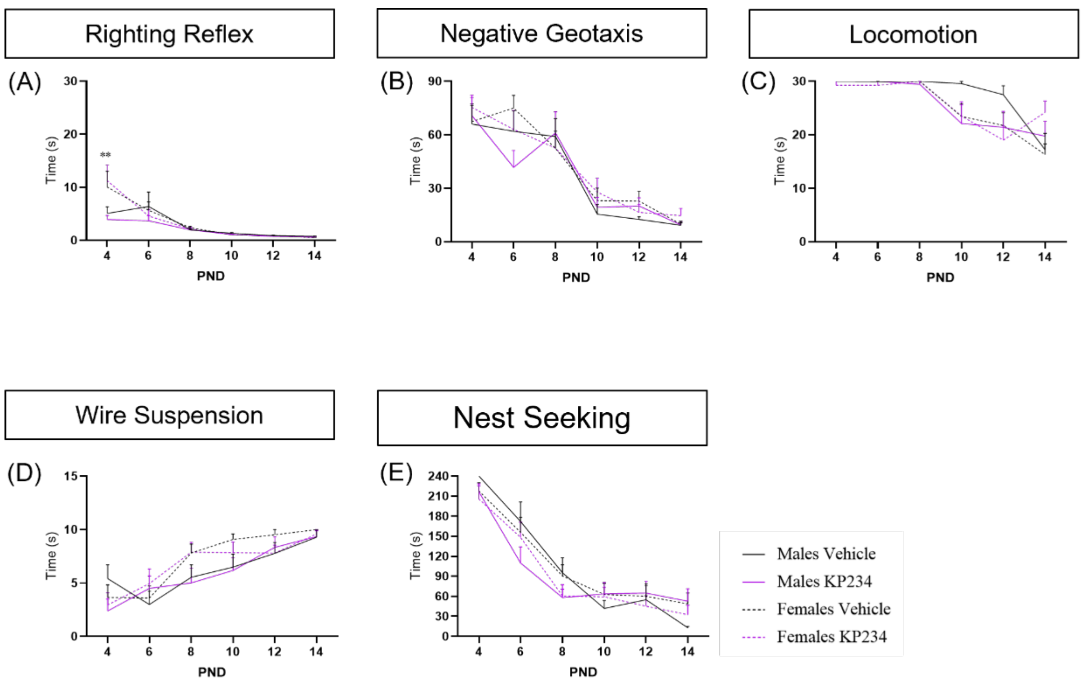

S 1 Developmental milestones

A) Righting Reflex: 3-way ANOVA mixed-effects revealed a significant PND effect (F=22.31, p-value<0.0001), significant sex effect (F=4.550, p-value=0.0340) and a significant interaction between sex and PND (F=4.393, p-value=0.0008), followed by Tukey's multiple comparisons test between treatment and sex groups for each PND. At PND4 for KP234 groups, the comparison between females and males showed a mean difference of 7.30 ± 1.680 (mean difference ± standard error), with a q-value=6.139 and a p-value=0.0048. **B) Negative geotaxis**: 3-way ANOVA mixed-effects revealed a significant PND effect (F=43.35, p-value<0.0001), followed by Tukey's multiple comparisons test, between treatment and sex groups for each PND (no significant differences). **C) Locomotion**: 3-way ANOVA mixed-effects revealed a significant PND effect (F=26.54, p-value<0.0001) and a significant interaction between treatment and PND (F=3.708, p-value=0.0030), followed by Tukey's multiple comparisons test between treatment and sex groups for each PND (no significant differences). **D) Wire Suspension**: 3-way ANOVA mixed-effects revealed

a significant PND effect (F=22.46, p-value<0.0001) and a significant sex effect (F=5.390, p-value=0.0211), followed by Tukey's multiple comparisons test between treatment and sex groups for each PND (no significant differences). **E) Nest seeking**: 3-way ANOVA mixed-effects revealed a significant PND effect (F=63.88, p-value<0.0001), followed by Tukey's multiple comparisons test, between treatment and sex groups for each PND (no significant differences). Sample size: males Veh = 10; males KP234 = 10; females Veh = 11; females KP234 = 11. Data represented as lines during time, error bars above the lines represent SEM; * represent differences between sexes in KP234 injected groups.

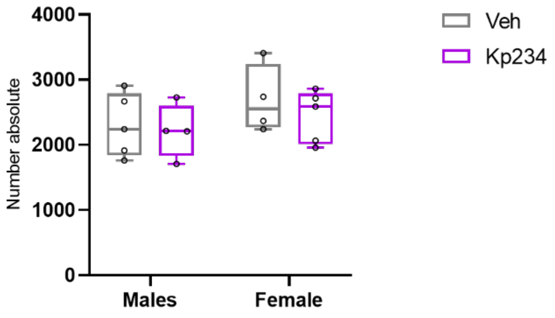

S 2 Number of calls at P29 during juvenile social play

Sample size: males Veh pairs = 5; males KP234 pairs = 4; females Veh pairs = 4; females KP234 pairs = 4. Data represented as box-plot min to máx.; circles represent pairs..

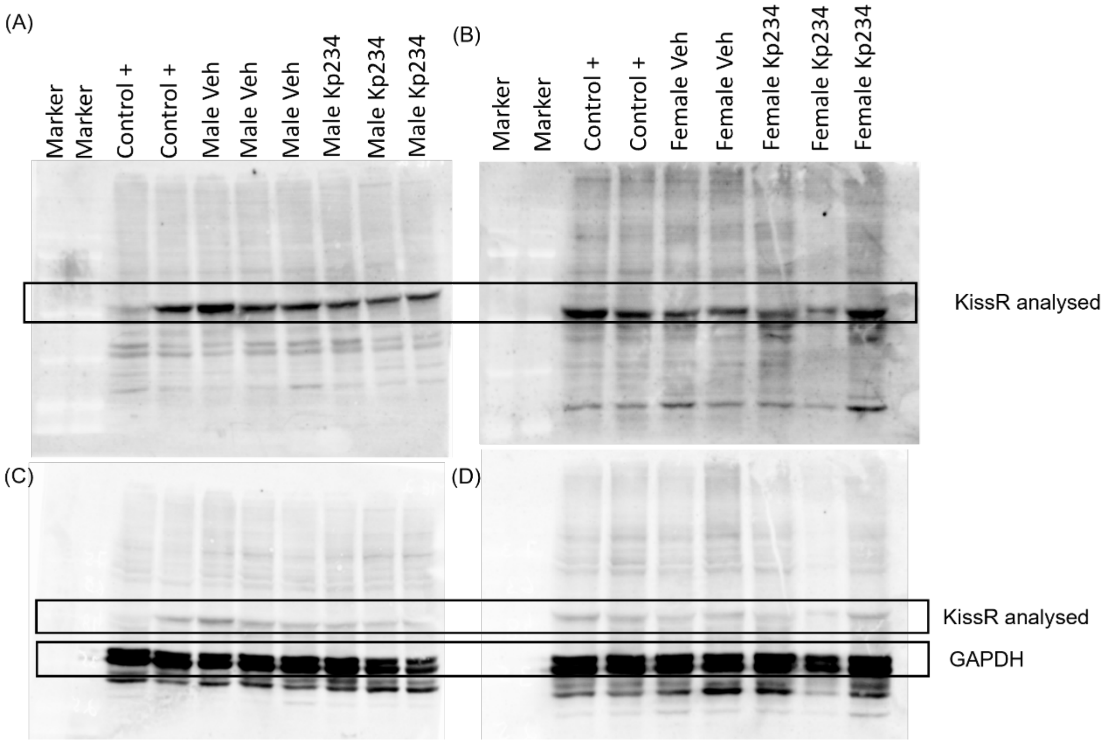

S 3 Representative membranes of Western blotting

A) Membrane 1 containing only males without housekeeping incubation B)Membrane containing only females without housekeeping incubation C) Membrane 1 containing only males with housekeeping incubation D)Membrane containing only females with housekeeping incubation
